# IFN-ε Is Constitutively Expressed by Cells of the Reproductive Tract and Is Inefficiently Secreted by Fibroblasts and Cell Lines

**DOI:** 10.1371/journal.pone.0071320

**Published:** 2013-08-09

**Authors:** Pascale Hermant, Cédric Francius, Frédéric Clotman, Thomas Michiels

**Affiliations:** 1 Université catholique de Louvain, de Duve Institute, Brussels, Belgium; 2 Université catholique de Louvain, Institute of Neuroscience, Brussels, Belgium; Kantonal Hospital St. Gallen, Switzerland

## Abstract

Type-I interferons (IFNs) form a large family of cytokines that primarily act to control the early development of viral infections. Typical type-I IFN genes, such as those encoding IFN-α or IFN-β are upregulated by viral infection in many cell types. In contrast, the gene encoding IFN-ε was reported to be constitutively expressed by cells of the female reproductive tract and to contribute to the protection against vaginal infections with herpes simplex virus 2 and *Chlamydia muridarum*. Our data confirm the lack of induction of IFN-ε expression after viral infection and the constitutive expression of IFN-ε by cells of the female but also of the male reproductive organs. Interestingly, when expressed from transfected expression plasmids in 293T, HeLa or Neuro2A cells, the mouse and human IFN-ε precursors were inefficiently processed and secretion of IFN-ε was minimal. Analysis of chimeric constructs produced between IFN-ε and limitin (IFN-ζ) showed that both the signal peptide and the mature moiety of IFN-ε contribute to poor processing of the precursor. Immunofluorescent detection of FLAG-tagged IFN-ε in transfected cells suggested that IFN-ε and chimeric proteins were defective for progression through the secretory pathway. IFN-ε did not, however, act intracellularly and impart an antiviral state to producing cells. Given the constitutive expression of IFN-ε in specialized cells and the poor processing of IFN-ε precursor in fibroblasts and cell lines, we hypothesize that IFN-ε secretion may require a co-factor specifically expressed in cells of the reproductive organs, that might secure the system against aberrant release of this IFN.

## Introduction

Type I interferons (IFNs), are a family of cytokines endowed with a potent antiviral activity [1]. Members of this family also referred to as IFN-α/β, include IFN-α, IFN-β, IFN-ε, IFN-κ, IFN-ω, IFN-τ (ovines and bovines) and IFN-ζ or limitin (mice). Type I IFNs are reported to bind a common heterodimeric receptor (IFNAR) [2], thereby eliciting a signal transduction cascade leading to the transcriptional activation of hundreds of interferon stimulated genes (ISGs) that contribute to antiviral activity [3–5].

The gene encoding IFN-ε was identified as a typical, single, intron-less type-I IFN gene, mapping to the IFN locus of human chromosome 9 or mouse chromosome 4 [6,7]. Although recent genetic analyses reveal frequent polymorphisms in the human *IFNE* gene [8], this gene is well conserved across mammals [6,9,10]. It has been shown that human IFN-ε can bind to the type I IFN receptor (IFNAR) [11] and possesses some antiviral activity [9,12,13].

Interestingly, a recent study by Fung et al. reports that, unlike other characterized type I IFN genes, the gene coding for IFN-ε was not transcriptionnally upregulated by treating cells with synthetic ligands that activate other type I IFN genes. Instead, IFN-ε was expressed in a tissue-specific fashion, by eptithelial cells of the female reproductive tract. IFN-ε was induced by estrogen administration, varied according to the estrous cycle, and was downregulated during pregnancy. Importantly, *Ifne*-deficient mice had increased susceptibility to vaginal infection by herpes simplex virus 2 and *Chlamydia muridarum* [10].

In this work, we confirm the constitutive expression of IFN-ε by cells of the female but also the male reproductive organs. We show that maturation and secretion of IFN-ε is inefficient in cell lines and fibroblasts, and we therefore hypothesize that IFN-ε secretion by cells of reproductive organs involves a specific co-factor lacking in other cells.

## Materials and Methods

### Animal experiments

Ethics statement: Handling of mice (agreement LA1230472) and experimental procedures were conducted in accordance with the EEC directive 86/609/CEE and the related Belgian law of April 6th 2010. The study and protocol used in this study were approved by the ethics committee of the University of Louvain under the agreement # 2010/UCL/MD/031.

### Cells, transfections, cell treatments

Cell lines used in this study were human 293T (kindly provided by F. Tangy, Pasteur Institute, Paris) [14] and HeLa epithelial cells (ATCC), mouse Neuro2A neuroblastoma (ECACC) and BALB/3T3 fibroblasts (kindly provided by Francis Brasseur, Ludwig Institute for cancer research, Brussels) [15]. Cells were grown in Dulbecco Modified Eagle medium (DMEM, Lonza ref 12-604F) containing ultraglutamine and 4.5 gr/L of glucose, and supplemented with 10% of fetal calf serum (Sigma) and 50 units/ml of penicillin/streptomycin (Lonza). Mouse embryonic fibroblasts (MEFs) were isolated from C57BL/6 mice by standard procedures. Briefly, embryos were harvested at day 14.5 of gestation. The head, heart, liver, intestine and kidneys were removed and the rest of the embryo was placed in a Petri dish containing Trypsin-EDTA (Lonza, 170 000 U/L Trypsin, 200 mg/L EDTA) in which the tissue was minced. After 13 minutes of incubation at 37° C, the tissue was homogenized by pipetting and centrifuged to eliminate undissociated tissue fragments. Cells were then grown in DMEM supplemented as above. MEFs were then immortalized by transduction of pPH51, a retroviral vector derived from pQCXIN (Stratagene) and expressing the simian virus 40 large T antigen. Immortalized MEFs were called MEFs/T.

Transfection of cells was performed using LT1 reagent (Mirus), according to the manufacturer’s instructions. For Brefeldin A treatment, GolgiPlug (ref 555029, BD Biosciences) was diluted 1000-fold in culture medium. IFN cytopathic effect reduction assay was performed as described in [16]. Relative antiviral activities were calculated as the highest dilution factor of the sample, which protected more than 50% of the cells against Mengo virus infection. Values are relative to those obtained for culture medium.

### Viruses and infections

KJ7 is a virus derived from Theiler’s murine encephalomyelitis virus (TMEV) DA1 strain. In this virus, the green fluorescent protein (GFP) coding region replaces codons 5 to 67 of the leader protein coding sequence. Mengo virus (a strain of encephalomyocarditis virus - EMCV) used in this study is an attenuated variant carrying a shortened polyC tract (24 C) in its 5' non-coding region. This virus was produced, as previously described [17] from the pMC24 plasmid carrying the full-length genome of the virus, cloned as cDNA [18]. Three six week-old male C57BL/6 Mx1^+/+^ mice were inoculated intraperitoneally with 10^6^ pfu of Mengo virus in 250 µl of phosphate buffered saline (PBS) and three mice were left untreated. Four days post-infection, mice were euthanized and perfused with PBS before organs harvest.

### Expression vectors

**Figure 1:**
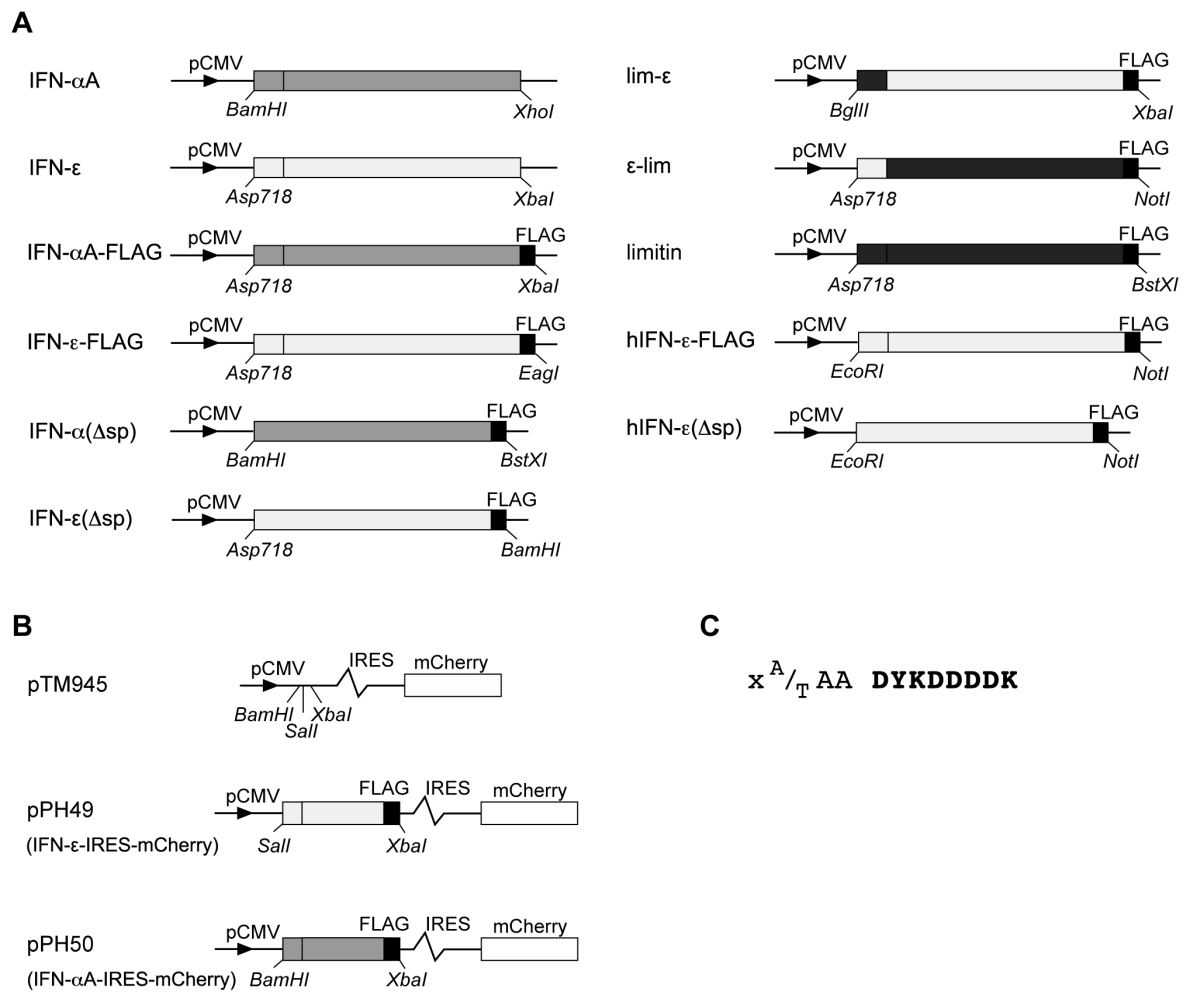
Plasmid constructs. A. pcDNA3 derivatives expressing FLAG-tagged or untagged IFNs. In these plasmids, IFN coding regions (frames) are cloned downstream of the cytomegalovirus promoter (pCMV). Restriction sites used for cloning IFN reading frames are shown. FLAG tag coding sequences were added downstream of the last IFN codon as indicated in C. IFN-αA(Δsp) and IFN-ε(Δsp) are constructs where the region encoding the signal peptide of the IFN precursor was deleted. lim-ε: chimeric IFN precursor with the signal peptide of limitin and the mature moiety of IFN-ε.ε-lim is the converse chimeric precursor with the signal peptide of IFN-ε and the mature moiety of limitin. Human IFN-ε with or without the signal peptide are indicated hIFN-ε and hIFN-ε(Δsp). Note that the various elements on these graphic representations are not to scale. B. Lentiviral vectors. In these vectors, transcription of the IFN gene is driven by the cytomegalovirus promoter. The IRES sequence from TMEV allows co-expression of the cloned coding sequence with the red fluorescent protein mCherry. C. Sequence of the IFN-FLAG junctions. X represents the last amino acid of IFN. The linker sequence between IFN and FLAG (bold letters) is AAA for ε-lim and limitin and TAA for the other constructs.

The coding region of the mouse *Ifne* gene was cloned in the pcDNA3 expression vector, downstream of a CMV promoter, as previously done for mouse IFN-αA and IFN-β [7,16]. Additional constructs were generated, encoding C-terminally FLAG-tagged IFNs. In the latter constructs, the FLAG sequence is separated from the last IFN amino acid by a three amino acid linker (Figure 1). Plasmids encoding FLAG-tagged IFNs were derived from pAGE1, a pcDNA3 derivative where a FLAG epitope coding sequence terminated by a stop codon was cloned between the *Not*I and *Xba*I sites, at the 3' end of the vector’s multicloning site. The sequence cloned between *Not*I and *Xba*I is 5'- GCG GCC GCA GAC TAC AAG GAC GAC GAT GAC AAG TGA ATC TAG A. This vector allows the expression of C-terminally FLAG-tagged proteins. Lentiviral vectors were derived from pCCLsin. PPT. hPGK. GFP.pre (kindly provided by Luigi Naldini, Ospedale San Raffaele - Milano, Italy) [19]. pTM945 was generated by inserting, in the backbone of this vector: a cytomegalovirus promoter, a multicloning site, an IRES from TMEV [20] and the mCherry coding sequence. ORFs of the murine IFNαA and IFN-ε were then subcloned in this plasmid using the *Sal*I/*Xba*I and *Bam*HI/*Xba*I restriction sites respectively. Expression vectors used in this study are presented in Figure 1.

### Western blot analysis

Total protein extracts were prepared by collecting and boiling the cells for 5 min in Laemmli buffer, 24h after transfection, or 30h after transfection in the case of Brefeldin A treatment. IFN-FLAG expression was analysed by Western blot using sodium dodecyl sulfate-polyacrylamide gel electrophoresis (SDS-PAGE) gels containing 15% acrylamide. The blot was probed with anti-FLAG M2 monoclonal antibody (Sigma-Aldrich F3165).

### Immunolabeling

Cells were fixed with 4% paraformaldehyde (PFA) in phosphate buffer saline (PBS) 24 hours post-transfection and permeabilized for 5 min with 0.1% triton X-100. FLAG-tagged IFNs were detected using an anti-FLAG mouse monoclonal antibody (Sigma-Aldrich, F3165, used at 1/1000) and a secondary antibody labeled with Alexafluor488 (Invitrogen, Life technologies ref A11029). The endoplasmic reticulum compartment was identified by co-transfection of pDsRed-ER [21]. The Golgi compartment was identified after staining glycosylated proteins with Alexafluor 594-conjugated Wheat Germ Agglutinin (WGA) (Molecular Probes, W11262).

### Quantitative RT-PCR

RNA was isolated from organs, reverse-transcribed and subjected to quantitative RT-PCR (RT-qPCR) as previously described [22], using SybrGreen and the MyIQ^TM^ apparatus (Biorad). Primer sequences were 5’-GCC GAA AGC CAC GTG TGT AA (sense) and 5’-AGA TCC CAG CCA GTG GGG TA (antisense) for Mengo virus, 5’-ATG AAC AAC AGG TGG ATC CTC C (sense) and 5’-AGG AGC TCC TGA CAT TTC CGA A (antisense) for IFN-β, 5'-GGA TGC CTG GGA GAG AAT CG-3' (sense) and 5'-TCG CCT GCT CTT CGA AAC TG-3' (antisense) for *Oasl2* and 5’-CGG TGT TGC TGC TCT TGG TT (sense), 5’-TCA CAG GCT GCT GAG GAA GC (antisense) for IFN-ε and 5'- AGA GGG AAA TCG TGC GTG AC-3' (sense) and 5'- CAA TAG TGA TGA CCT GGC CGT-3' (antisense) for β-actin. Standards consisted of 10-fold dilutions of known concentrations of plasmids carrying the corresponding DNA sequences: pMC24 (Mengo virus), pcDNA3-IFN-β, pCS40 (Oasl2) pcDNA3-IFN-ε, or pTM793 (β-actin).

### Flow cytometry

Adherent cells were trypsinized and resuspended in phosphate-buffered saline containing 5% of filtered fetal calf serum and 1% of paraformaldehyde. Data acquisition was performed on a LSR Fortessa cell analyzer (BD biosciences) using the FACSDiva software. Analysis was done using the FlowJo software. Cells were gated according to size (forward and side scatter) before analysis for GFP and mCherry fluorescence.

### Statistical analysis

Data were analyzed with Prism version 4.0c using one-tailed Mann–Whitney *U* test. *P* values ≤ 0.05 were considered significant.

## Results

### Expression of IFN-ε *in vivo*

**Figure 2:**
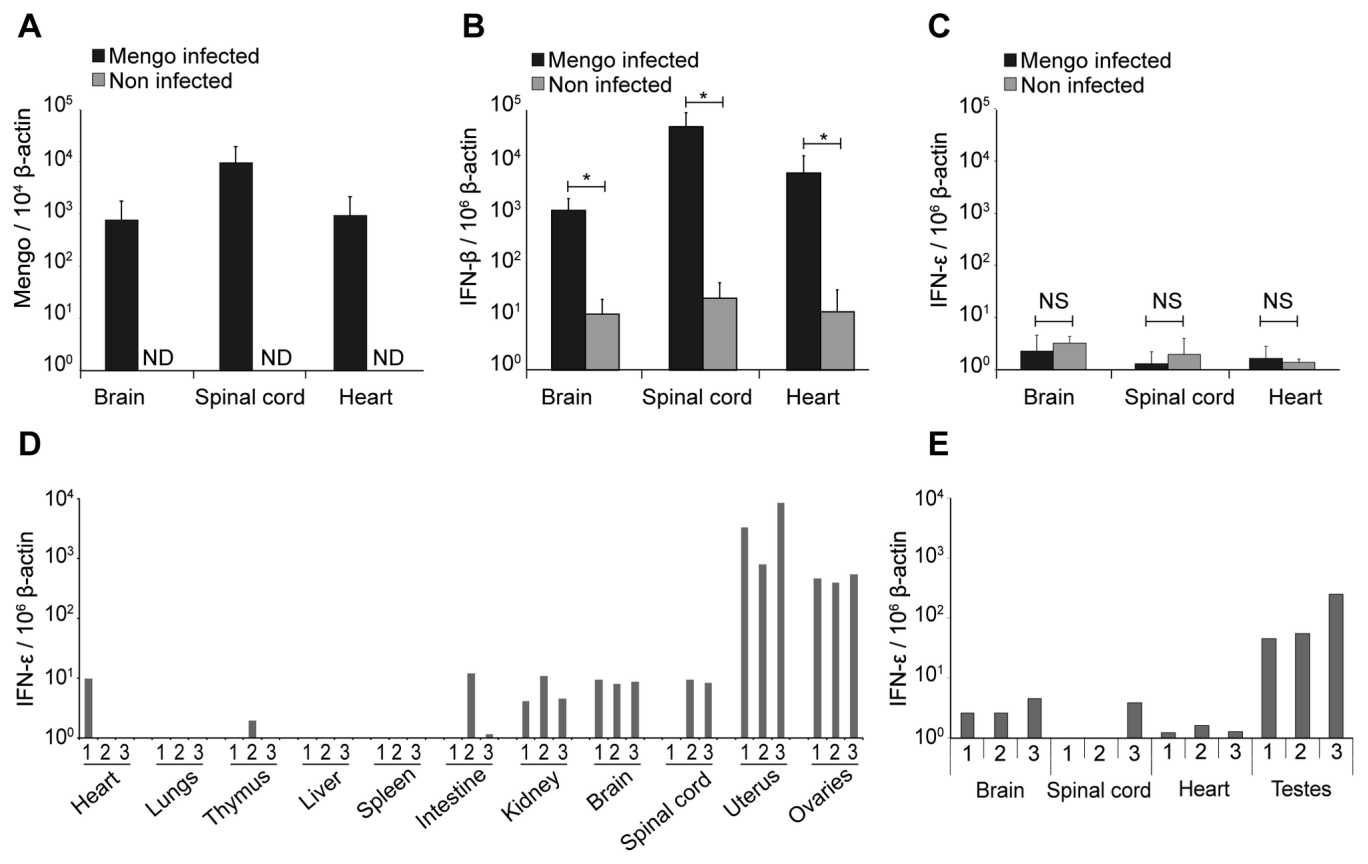
In vivo expression of IFN-ε. A–C. RT-qPCR analysis of Mengo virus replication (A), *Ifnb* expression (B) and *Ifne* expression (C) in the brain, spinal cord and heart of Mengo virus-infected mice. Histograms show the mean ± SD of Mengo virus cDNA copies per 10^4^ β-actin cDNA copies (n=3). ND: not detected. NS: non significant. D–E. RT-qPCR data showing the expression of *Ifne* in organs collected from uninfected female (D) and male (E) C57BL/6 mice. Each column refers to an individual sample and indicates the number of *Ifne* cDNA copies per 10^6^ β-actin cDNA copies.

We used quantitative RT-PCR analysis to address whether *Ifne* expression could be upregulated *in vivo*, after viral infection. Therefore, mRNA expression levels of the genes coding for IFN-β and IFN-ε were measured in mice, 4 days after intraperitoneal inoculation of Mengo virus. In the brain, spinal cord and heart, the organs that are the most infected by this virus, *Ifnb* expression was clearly upregulated but *Ifne* expression was not (Figure 2 A, B, C). These data confirm, with another infection model, the lack of upregulation of *Ifne* transcription by viral infection that was observed in previous studies [10,23].

As *Ifne* was not upregulated by viral infection, we measured the expression of the *Ifne* gene in various tissues of uninfected male and female mice. The results presented in Figure 2D show constitutive expression of *Ifne* in the uterus and ovaries of female mice, in agreement with the data of Fung et al. [10]. In addition, we detected higher levels of *Ifne* transcription in testes than in other organs of male mice (Figure 2E). Consistent with the data of Fung et al., we detected a slightly higher expression of *Oasl2*, a strongly inducible IFN-stimulated gene (ISG), in the uterus of female mice, as well as in ovaries (Supplementary Figure S1). The levels of *Oasl2* expression in these organs correlated with those of *Ifne*, suggesting that IFN-ε is produced locally. We also detected higher *Oasl2* mRNA expression in the intestine although IFN-ε was not expressed in this organ. We do not know the reason for this. It might be the consequence of homeostatic IFN expression triggered by the microbiota in this organ [24].

Taken together, our data largely confirm the recent work of Fung et al. showing that IFN-ε is not induced by viral infection but is constitutively expressed in reproductive tissues, and extend the observation to the male reproductive tissue [10].

### Expression of IFN-ε by transfected cells

**Figure 3:**
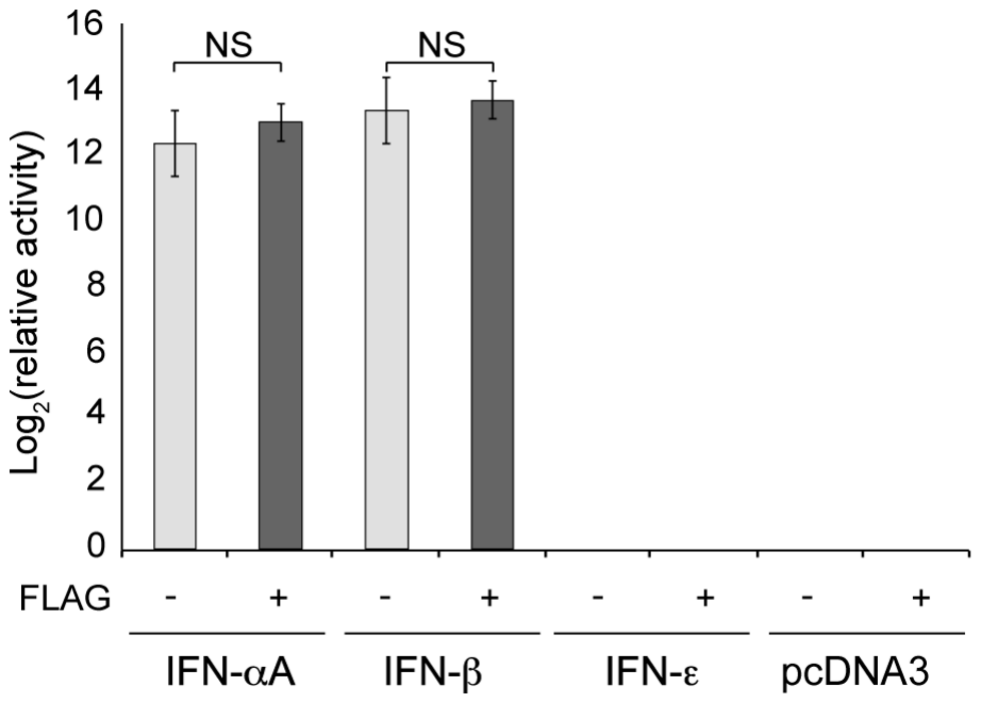
Activity of FLAG-tagged and untagged mouse IFNs. Histograms show the log_2_ of antiviral activities detected in the supernatant of Neuro2A cells collected 24h after transfection of the pcDNA3 derivatives expressing the indicated FLAG-tagged (+) or untagged (-) IFNs or after transfection of the corresponding empty vectors (pcDNA3). Antiviral activities are presented relative to those of culture medium. NS: non significant (Mann–Whitney *U* test).

To assess IFN-ε antiviral activity, 293T cells were transfected with expression plasmids coding for IFN-α, IFN-β, IFN-ε or with equivalent constructs coding for C-terminally FLAG-tagged IFNs. Supernatants of transfected cells, collected 24 and 48 hours post-transfection were assayed for antiviral activity using a cytopathic effect reduction assay. Antiviral activity of tagged and untagged IFN-αA and IFN-β did not differ significantly (Figure 3), suggesting that the C-terminal FLAG epitope affected neither IFN production nor receptor binding. Surprisingly, little, if any, antiviral activity was detected in the supernatant of 293T cells transfected with vectors expressing tagged or untagged IFN-ε (Figure 3). Similar results were observed when IFN expressing plasmids were transfected in Neuro2A or BALB/3T3 cells that are of murine origin. We conclude that either IFN-ε has little antiviral activity or that this IFN was not expressed or not secreted by transfected cells.

### Processing of the IFN precursor

**Figure 4:**
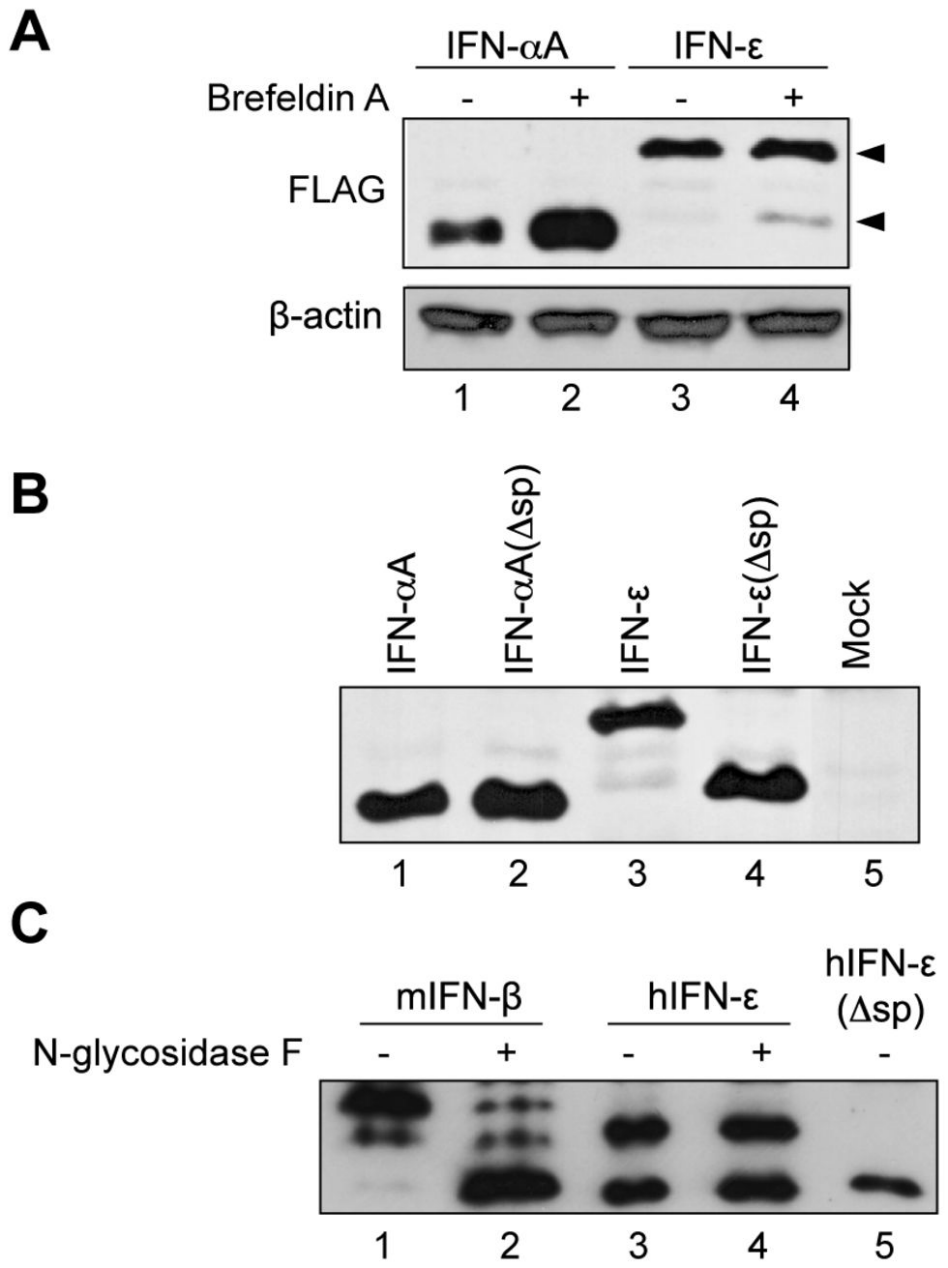
Processing of the IFN-ε precursor in transfected cells. Western blot analysis of IFN-α and IFN-ε processing in total protein extracts of Neuro2A cells transfected for 24h with pcDNA3 derivatives expressing FLAG tagged IFNs. A. Mouse IFN-αA and IFN-ε detection in the presence or absence of brefeldin A. Arrowheads point to the two bands detected for IFN-ε.β-actin detection was used as loading control. B. Detection of mouse IFN-αA and IFN-ε along with corresponding proteins expressed without signal peptide (Δsp). C. Human IFN-ε and mouse IFN-β detection in 293T cells before and after treatment with N-glycosidase F.

Western blot analysis was thus used to detect FLAG-tagged IFN-ε and IFN-αA in Neuro2A cells transfected with the expression vectors. Cells were either treated or mock-treated for 6 hours with brefeldin A prior to protein extraction, to trigger the retention of secreted proteins. As shown in Figure 4A, FLAG-IFN-ε was readily detected in extracts of transfected cells. Surprisingly, in contrast to IFN-αA, which migrated with an expected apparent molecular mass (19 kDa) and accumulated after brefeldin A treatment (Figure 4A, lanes 1 and 2), IFN-ε appeared as a major band migrating slower than expected (22 kDa) whose amount was not affected by brefeldin A treatment. A minor band migrating with an expected velocity (20 kDa) appeared after brefeldin A treatment (Figure 4A, lanes 3 and 4). These data suggest that the IFN-ε precursor is not properly processed in transfected cells. We confirmed that the minor band detected for IFN-ε and the major band detected for IFN-αA had molecular masses compatible with the mature forms of these IFNs by comparing their migration profiles with those of corresponding IFNs expressed without signal sequence (Figure 4B).

Interestingly, processing of human IFN-ε was also aberrant (Figure 4C). In contrast to mouse IFN-ε, human IFN-ε carries two putative N-glycosylation sites [13]. In extracts from transfected 293T cells, FLAG-tagged human IFN-ε was detected as two bands (Figure 4C, lane 3). Neither of the two bands corresponded to N-glycosylated IFN since N-glycosidase F treatment failed to modify the migration pattern (Figure 4C, lane 4). The upper band likely corresponded to the IFN-ε precursor. The lower band migrated with a velocity close to that of IFN-ε expressed without signal sequence (Figure 4C, lane 5). However, the fact that this IFN-ε form lacks N-glycosylation indicates that this IFN may not have reached the secretory pathway. FLAG-tagged murine IFN-β, taken as a control in this experiment, migrated as multiple bands (Figure 4C, lane 1) as expected from the fact that this IFN subtype carries three N-glycosylation sites [25]. After N-glycosidase F treatment of FLAG-IFN-β, a predominant band appeared at the expected molecular mass for the mature protein (Figure 4C, lane 2). These results show that the processing of the IFN-ε signal sequence is very inefficient in cells transfected with expression plasmids. In agreement with the above data, prediction for the presence of a signal peptide by the Signal P 4.1 server [26] was poor for mouse and human IFN-ε precursors, in contrast to other mouse type-I IFN precursors (Table 1).

**Table 1:** Signal peptide prediction (Signal P 4.1 Server).

**Interferon**	**Cleavage site^1^**	**D-score^2^**
**mouse IFN-αA**	**23-24**	**0.833 (Yes)**
**mouse IFN-β**	**21-22**	**0.882 (Yes)**
**mouse Limitin**	**21-22**	**0.561 (Yes)**
**mouse IFN-ε**	**21-22**	**0.519 (Yes)**
**human IFN-ε**	**21-22**	**0.328 (No)**

^1^ Position predicted for the cleavage site is between the indicated amino acids of the precursor protein.

^2^ D-score reflects the likelyhood of a signal peptide. Yes or No indicates whether a signal sequence is predicted by the server (D-score cutoff = 0.5).

**Figure 5:**
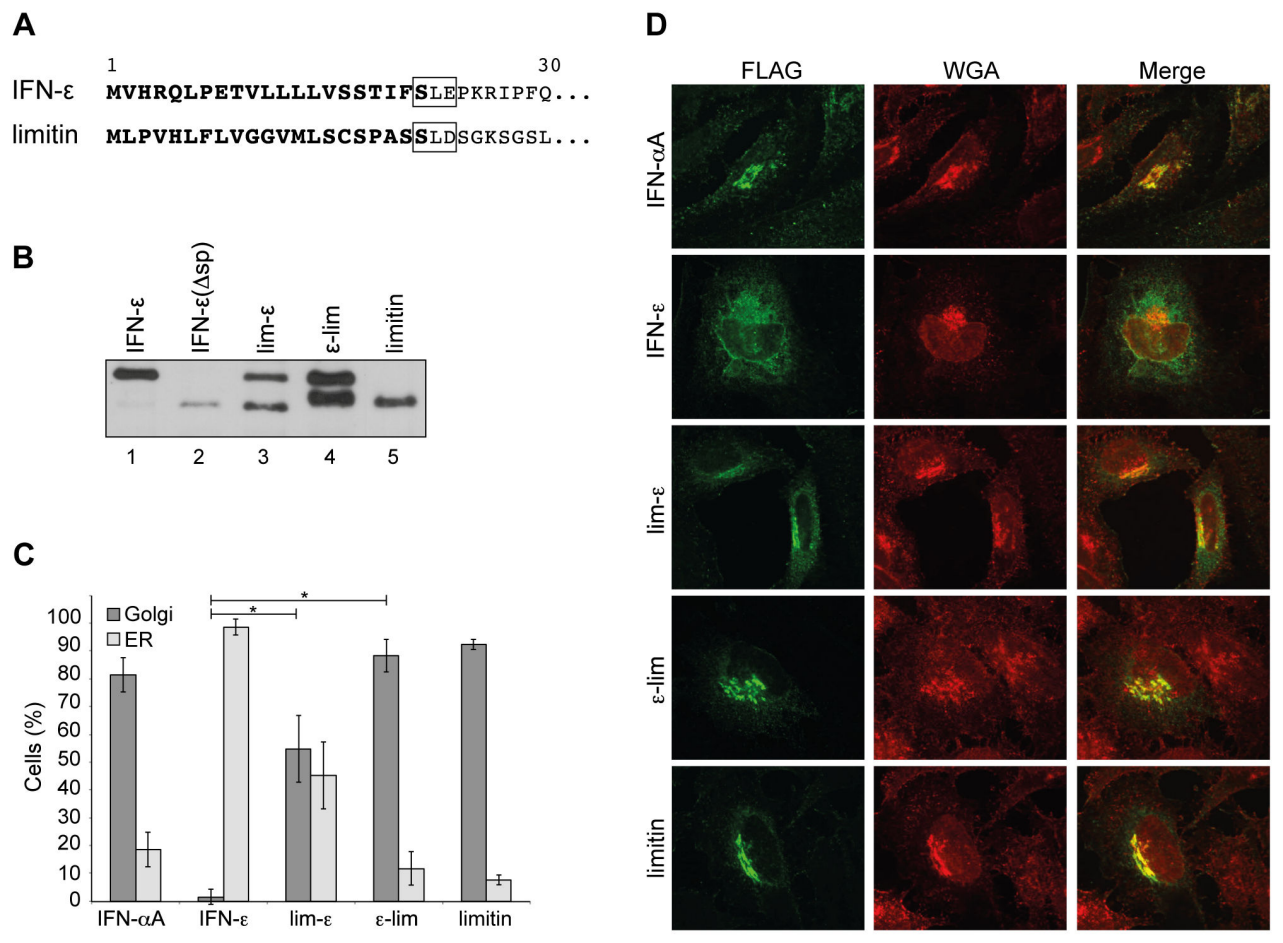
IFN-ε signal peptide is not fully functional. A. Signal peptides predicted for IFN-ε and limitin. Predicted signal peptides are indicated in bold letters. Related amino acids around the putative cleavage site are framed. B. Western blot analysis of cell lysates from Neuro2A cells that were transfected for 24h with pcDNA3 derivatives expressing FLAG-tagged IFN-ε, IFN-ε(Δsp), lim-ε, ε-lim or limitin. Cells were harvested in laemmli buffer twenty-four hours post-transfection. C. Histograms showing, for the indicated constructs, the proportion of cells where IFN colocalizes mostly with the Golgi or with the endoplasmic reticulum. Means and SD of countings from 4 immunostainings. For each counting, n = ± 200 for IFN-α, lim-ε, limitin and ε-lim; n = 100 for IFN-ε. D. Immunofluorescent detection of FLAG-tagged IFNs in HeLa cells transfected with plasmids expressing the indicated tagged IFNs. IFNs appear in green. The WGA lectin was used to detect glycosylated proteins and to highlight the Golgi network (red).

We thus asked whether this inefficient processing of the IFN-ε precursor was due to the sequence of the signal peptide. Limitin, also called IFN-ζ, is a type-I IFN displaying antiviral activity [27]. This IFN subtype was shown to be secreted efficiently from 293T cells transfected with an expression plasmid [7]. Yet, the amino acid sequence around the predicted cleavage site of the limitin precursor [28] is close to that predicted for IFN-ε (Figure 5A).

To test the influence of the signal peptide on IFN processing and secretion, we constructed chimeric plasmids by exchanging the signal peptide coding sequences of limitin and IFN-ε in the FLAG-tagged constructs (Figure 1). These constructs were transfected along with control plasmids in Neuro2A cells. As shown on Figure 5B, IFN-ε signal peptide replacement by that of limitin improved the processing of IFN-ε (Figure 5B, compare lanes 1 and 3). Conversely, when the limitin signal peptide was replaced with that of IFN-ε, a band appeared on western blots, compatible with immature limitin (Figure 5B, compare lanes 5 and 4). The same results were observed in 293T and HeLa cells (data not shown). These data suggest that both the signal sequence and the mature moiety of IFN-ε contribute to poor processing of the precursor.

### Progression of IFN through the secretory pathway

Next we used immunofluorescent labeling of the FLAG-tagged IFNs in transfected cells to test whether poor signal peptide processing correlated with altered progression of IFN in the secretory pathway. Therefore, plasmids encoding FLAG-tagged IFN-α, IFN-ε, lim-ε, ε-lim and limitin were transiently expressed in HeLa cells. Cells were observed by fluorescent microscopy in a blind experiment. Counting was done according to the main localisation of IFN, either in the Golgi compartment or in the endoplasmic reticulum (Figure 5C). While IFN-αA and limitin were mainly detected in the Golgi compartment, IFN-ε displayed a more diffuse localization in the cells, partly co-localizing with the endoplasmic reticulum (ER) and partly showing a more diffuse cytoplasmic pattern (Figure 5D). Only few cells showed IFN-ε associated with the Golgi compartment. It is noteworthy that IFN-ε detection was much weaker than detection of other IFNs, suggesting that part of the expressed IFN-ε was degraded in cells. Replacing the IFN-ε signal peptide by that of limitin significantly increased the detection of the chimeric protein in the Golgi compartment (one-tailed Mann Whitney test, p = 0.0143). Replacing the mature moiety of IFN-ε by that of limitin also increased the targeting of the chimeric protein to the Golgi compartment (one-tailed Mann Whitney test, p = 0.0143). These data confirm that both the signal sequence and the mature moiety of IFN-ε contribute to poor processing of the precursor protein and therefore prevent access of IFN-ε to the secretory pathway. Similar results were obtained in C57BL/6 mouse embryonnic fibroblasts (Supplementary Figure S2).

### Intracellular activity of IFN-ε

**Figure 6:**
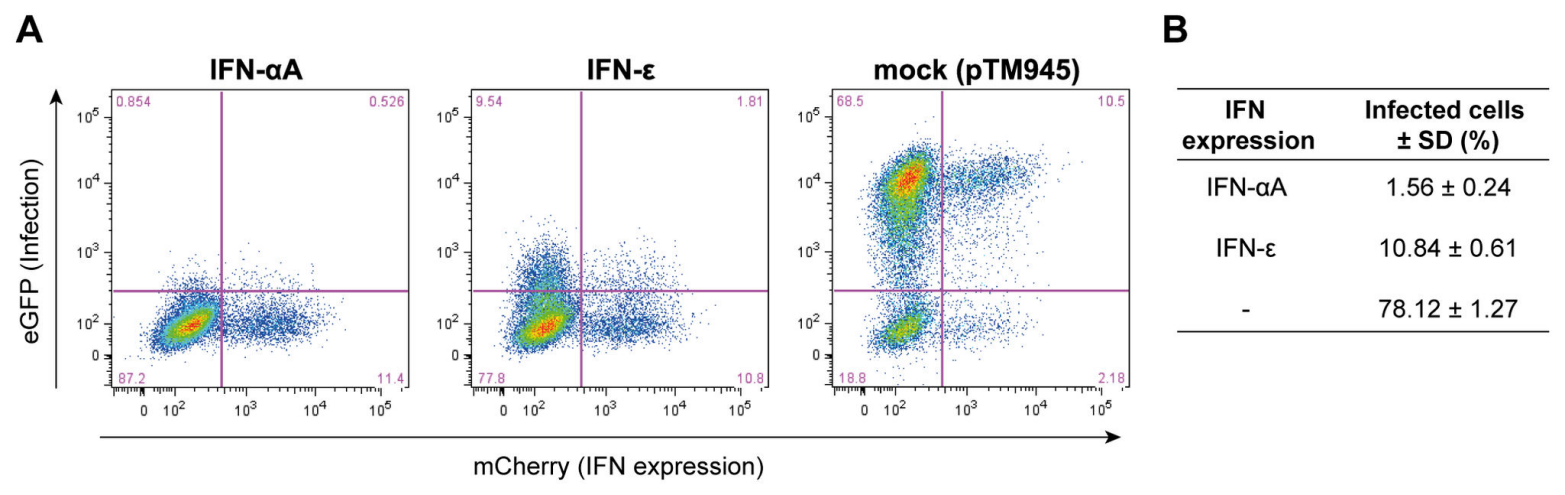
IFN-ε does not act intracellularly. FACS analysis of cells transduced for 4 days with bicistronic lentiviral constructs co-expressing mCherry and the indicated IFN. pTM945 is the empty vector expressing mCherry alone. A. Representative FACS dot plot. B. Table showing the mean and standard deviation of the percentage of infected cells in the total cell population (data from 3 infection experiments).

Since IFN-ε was inefficiently secreted from cells, we next asked whether IFN-ε could impart viral protection to producing cells, in the absence of secretion. Therefore, we transduced cells with lentiviral bicistronic vectors allowing the co-expression of the fluorescent protein mCherry and of murine IFN-αA (pPH50) or IFN-ε (pPH49), or with the empty vector expressing mCherry alone (pTM945) (Figure 6). Three days after transduction, the antiviral state of the transduced cell populations was analyzed by FACS, after infection with KJ07, a derivative of Theiler’s murine encephalomyelitis virus expressing eGFP. In this case, IFN-ε antiviral activity was detected but was low as compared to that of IFN-αA. For cells transduced with similar efficiencies, the percentage of infected cells was 10.84 ± 0.61% for the cells expressing IFN-ε and 1.56 ± 0.24% for the cells expressing IFN-αA (Figure 6). Interestingly, cells expressing IFN-ε, as detected by mCherry fluorescence, were not more protected against viral infection than untransduced mCherry-negative cells of the same population (Infection rates were 13.62% in IFN-ε expressing cells and 10.43% in IFN-ε negative cells). Thus, intracellular IFN-ε expression did not trigger resistance to viral infection.

## Discussion

Our data confirm the unusual features of the *Ifne* gene, i.e. that this gene is not transcriptionnally upregulated after viral infection and that it is constitutively expressed by cells of the female reproductive tract. Our data extend these observations to cells of the male reproductive tract. Recently, Fung et al. [10] demonstrated that, unlike other type-I IFNs, IFN-ε is hormonally regulated. Indeed, mouse *Ifne* expression was upregulated after estrogen administration. Accordingly, human *IFNE* expression in epithelial cells isolated from uterine endometrium was higher in the proliferative phase, when estrogen levels are the highest [10]. Moreover, progesterone receptor binding sites were identified in the promoters of both mouse and human IFN-ε genes [6]. Thus, regulation of IFN-ε expression strikingly differs from that of other type-I IFNs.

Type-I IFNs are secreted by most cell types and exert their antiviral activity on neighbouring cells. Surprisingly, we barely detected antiviral activity in the supernatant of cells transfected with vectors expressing IFN-ε. This led us to investigate whether this IFN was secreted by these cells. Interestingly, mouse and human IFN-ε precursors were inefficiently processed in cells transfected with expression vectors and secretion of IFN-ε was minimal. Analysis of chimeric constructs produced between IFN-ε and limitin (IFN-ζ) showed that both the signal peptide and the mature moiety of IFN-ε contribute to poor processing of the precursor. Immunofluorescent detection of FLAG-tagged IFN-ε in transfected HeLa cells or mouse embryonic fibroblasts suggested that IFN-ε progression through the secretory pathway was limited as the protein was rarely detected in the Golgi apparatus.

Given the constitutive expression of IFN-ε in specialized cells and poor processing of IFN-ε precursor in tested cell lines, we hypothesize that IFN-ε secretion may require a co-factor, such as a chaperone, specifically expressed in cells of the reproductive organs. On one hand, the requirement for a specific co-factor would secure the system against leaky production of IFN-ε in other tissues. On the other hand, a specific co-factor might act to regulate the secretion of IFN-ε in response to environmental triggers such as hormones or cytokines. Further experiments will be necessary to confirm this hypothesis.

In conclusion, our study highlights an unusual expression pattern and restriction in the secretion of a member of the type-I IFN family: IFN-ε. We demonstrate that this IFN is poorly secreted after transfection of an expression vector in different cell lines. As this IFN is constitutively expressed in cells of the female and male reproductive tracts, we postulate that IFN-ε secretion might be regulated by a specific factor expressed in these cells.

## Supporting Information

Figure S1 ***Oasl2* expression level is elevated in uterus and ovaries.**RT-qPCR data showing the expression of *Oasl2* in organs collected from uninfected female C57BL/6 mice (same as in Fig 2A). Each column refers to an individual sample and indicates the number of *Oasl2* cDNA copies per 10^2^ β-actin cDNA copies.
(TIF)

Figure S2 **Poor progression of IFN-ε through the secretory pathway of transfected MEFs/T.** A. Immunofluorescent detection of FLAG-tagged IFNs in MEFs/T cells transfected with plasmids expressing the indicated tagged IFNs. B. Histograms showing, for the indicated constructs, the proportion of cells where IFN colocalizes mostly with the Golgi (dark grey) or with the endoplasmic reticulum (light grey). The amounts of counted cells are indicated below each plot.
(TIF)

Materials and Methods S1 (DOCX)

## References

[1] Isaacs A, Lindenmann J (1957) Virus interference. I. Interferon Proc R Soc Lond B Biol Sci 147: 258-267. doi:10.1098/rspb.1957.0048. 26297790

[2] Uzé G, Schreiber G, Piehler J, Pellegrini S (2007) The receptor of the type I interferon family. Curr Top Microbiol Immunol 316: 71-95. doi:10.1007/978-3-540-71329-6_5. PubMed: 17969444. 17969444 10.1007/978-3-540-71329-6_5

[3] Liu SY, Sanchez DJ, Cheng G (2011) New developments in the induction and antiviral effectors of type I interferon. Curr Opin Immunol 23: 57-64. doi:10.1016/j.coi.2010.11.003. PubMed: 21123041. 21123041 10.1016/j.coi.2010.11.003 PMC3822007

[4] Randall RE, Goodbourn S (2008) Interferons and viruses: an interplay between induction, signalling, antiviral responses and virus countermeasures. J Gen Virol 89: 1-47. doi:10.1099/vir.0.83391-0. PubMed: 18089727. 18089727 10.1099/vir.0.83391-0

[5] Schoggins JW, Wilson SJ, Panis M, Murphy MY, Jones CT et al. (2011) A diverse range of gene products are effectors of the type I interferon antiviral response. Nature 472: 481-485. doi:10.1038/nature09907. PubMed: 21478870. 21478870 10.1038/nature09907 PMC3409588

[6] Hardy MP, Owczarek CM, Jermiin LS, Ejdebäck M, Hertzog PJ (2004) Characterization of the type I interferon locus and identification of novel genes. Genomics 84: 331-345. doi:10.1016/j.ygeno.2004.03.003. PubMed: 15233997. 15233997 10.1016/j.ygeno.2004.03.003

[7] van Pesch V, Lanaya H, Renauld JC, Michiels T (2004) Characterization of the murine alpha interferon gene family. J Virol 78: 8219-8228. doi:10.1128/JVI.78.15.8219-8228.2004. PubMed: 15254193. 15254193 10.1128/JVI.78.15.8219-8228.2004 PMC446145

[8] Manry J, Laval G, Patin E, Fornarino S, Itan Y et al. (2011) Evolutionary genetic dissection of human interferons. J Exp Med 208: 2747-2759. doi:10.1084/jem.20111680. PubMed: 22162829. 22162829 10.1084/jem.20111680 PMC3244034

[9] Sang Y, Rowland RR, Hesse RA, Blecha F (2010) Differential expression and activity of the porcine type I interferon family. Physiol Genomics 42: 248-258. doi:10.1152/physiolgenomics.00198.2009. PubMed: 20406849. 20406849 10.1152/physiolgenomics.00198.2009

[10] Fung KY, Mangan NE, Cumming H, Horvat JC, Mayall JR et al. (2013) Interferon-epsilon protects the female reproductive tract from viral and bacterial infection. Science 339: 1088-1092. doi:10.1126/science.1233321. PubMed: 23449591. 23449591 10.1126/science.1233321 PMC3617553

[11] Huang J, Smirnov SV, Lewis-Antes A, Balan M, Li W et al. (2007) Inhibition of type I and type III interferons by a secreted glycoprotein from Yaba-like disease virus. Proc Natl Acad Sci U S A 104: 9822-9827. doi:10.1073/pnas.0610352104. PubMed: 17517620. 17517620 10.1073/pnas.0610352104 PMC1887573

[12] Day SL, Ramshaw IA, Ramsay AJ, Ranasinghe C (2008) Differential effects of the type I interferons alpha4, beta, and epsilon on antiviral activity and vaccine efficacy. J Immunol 180: 7158-7166. PubMed: 18490714. 18490714 10.4049/jimmunol.180.11.7158

[13] Peng FW, Duan ZJ, Zheng LS, Xie ZP, Gao HC et al. (2007) Purification of recombinant human interferon-epsilon and oligonucleotide microarray analysis of interferon-epsilon-regulated genes. Protein Expr Purif 53: 356-362. doi:10.1016/j.pep.2006.12.013. PubMed: 17287131. 17287131 10.1016/j.pep.2006.12.013

[14] DuBridge RB, Tang P, Hsia HC, Leong PM, Miller JH et al. (1987) Analysis of mutation in human cells by using an Epstein-Barr virus shuttle system. Mol Cell Biol 7: 379-387. PubMed: 3031469. 3031469 10.1128/mcb.7.1.379-387.1987 PMC365079

[15] Aaronson SA, Todaro GJ (1968) Development of 3T3-like lines from Balb-c mouse embryo cultures: transformation susceptibility to SV40. J Cell Physiol 72: 141-148. doi:10.1002/jcp.1040720208. PubMed: 4301006. 4301006 10.1002/jcp.1040720208

[16] van Pesch V, Michiels T (2003) Characterization of interferon-alpha 13, a novel constitutive murine interferon-alpha subtype. J Biol Chem 278: 46321-46328. doi:10.1074/jbc.M302554200. PubMed: 12930842. 12930842 10.1074/jbc.M302554200

[17] Michiels T, Dejong V, Rodrigus R, Shaw-Jackson C (1997) Protein 2A is not required for Theiler’s virus replication. J Virol 71: 9549-9556. PubMed: 9371618. 9371618 10.1128/jvi.71.12.9549-9556.1997 PMC230262

[18] Duke GM, Osorio JE, Palmenberg AC (1990) Attenuation of Mengo virus through genetic engineering of the 5' noncoding poly(C) tract. Nature 343: 474-476. doi:10.1038/343474a0. PubMed: 2153940. 2153940 10.1038/343474a0

[19] Follenzi A, Ailles LE, Bakovic S, Geuna M, Naldini L (2000) Gene transfer by lentiviral vectors is limited by nuclear translocation and rescued by HIV-1 pol sequences. Nat Genet 25: 217-222. doi:10.1038/76095. PubMed: 10835641. 10835641 10.1038/76095

[20] Shaw-Jackson C, Michiels T (1999) Absence of internal ribosome entry site-mediated tissue specificity in the translation of a bicistronic transgene. J Virol 73: 2729-2738. PubMed: 10074119. 10074119 10.1128/jvi.73.4.2729-2738.1999 PMC104029

[21] Roderick HL, Campbell AK, Llewellyn DH (1997) Nuclear localisation of calreticulin in vivo is enhanced by its interaction with glucocorticoid receptors. FEBS Lett 405: 181-185. doi:10.1016/S0014-5793(97)00183-X. PubMed: 9089287. 9089287 10.1016/s0014-5793(97)00183-x

[22] Paul S, Michiels T (2006) Cardiovirus leader proteins are functionally interchangeable and have evolved to adapt to virus replication fitness. J Gen Virol 87: 1237-1246. doi:10.1099/vir.0.81642-0. PubMed: 16603526. 16603526 10.1099/vir.0.81642-0

[23] Delhaye S, Paul S, Blakqori G, Minet M, Weber F et al. (2006) Neurons produce type I interferon during viral encephalitis. Proc Natl Acad Sci U S A 103: 7835-7840. doi:10.1073/pnas.0602460103. PubMed: 16682623. 16682623 10.1073/pnas.0602460103 PMC1458506

[24] Ganal SC, Sanos SL, Kallfass C, Oberle K, Johner C et al. (2012) Priming of natural killer cells by nonmucosal mononuclear phagocytes requires instructive signals from commensal microbiota. Immunity 37: 171-186. doi:10.1016/j.immuni.2012.05.020. PubMed: 22749822. 22749822 10.1016/j.immuni.2012.05.020

[25] Sommereyns C, Michiels T (2006) N-glycosylation of murine IFN-beta in a putative receptor-binding region. J Interferon Cytokine Res 26: 406-413. doi:10.1089/jir.2006.26.406. PubMed: 16734561. 16734561 10.1089/jir.2006.26.406

[26] Petersen TN, Brunak S, von Heijne G, Nielsen H (2011) SignalP 4.0: discriminating signal peptides from transmembrane regions. Nat Methods 8: 785-786. doi:10.1038/nmeth.1701. PubMed: 21959131. 21959131 10.1038/nmeth.1701

[27] Kawamoto S, Oritani K, Asada H, Takahashi I, Ishikawa J et al. (2003) Antiviral activity of limitin against encephalomyocarditis virus, herpes simplex virus, and mouse hepatitis virus: diverse requirements by limitin and alpha interferon for interferon regulatory factor 1. J Virol 77: 9622-9631. doi:10.1128/JVI.77.17.9622-9631.2003. PubMed: 12915574. 12915574 10.1128/JVI.77.17.9622-9631.2003 PMC187381

[28] Oritani K, Kincade PW, Zhang C, Tomiyama Y, Matsuzawa Y (2001) Type I interferons and limitin: a comparison of structures, receptors, and functions. Cytokine Growth Factor Rev 12: 337-348. doi:10.1016/S1359-6101(01)00009-0. PubMed: 11544103. 11544103 10.1016/s1359-6101(01)00009-0

