# Supplementary figures and images for: IFN-ε Is Constitutively Expressed by Cells of the Reproductive Tract and Is Inefficiently Secreted by Fibroblasts and Cell Lines
Source: PLoS One. 2013 Aug 9;8(8):e71320. doi: 10.1371/journal.pone.0071320 (PMC3739789; doi:10.1371/journal.pone.0071320)

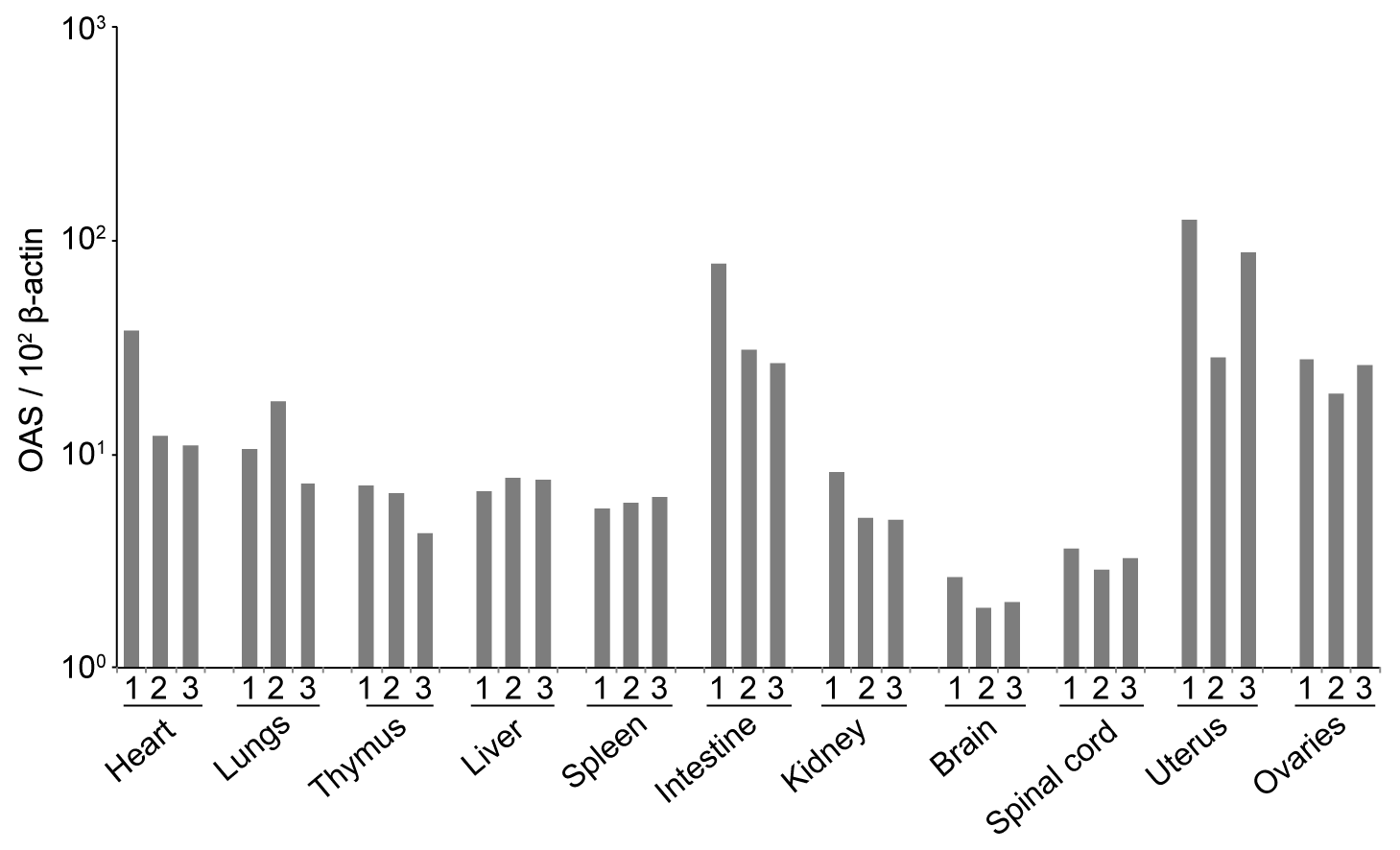

Supplement: Figure S1 — Oasl2 expression level is elevated in uterus and ovaries.RT-qPCR data showing the expression of Oasl2 in organs collected from uninfected female C57BL/6 mice (same as in Fig 2A). Each column refers to an individual sample and indicates the number of Oasl2 cDNA copies per 102 β-actin cDNA copies. (TIF) [file pone.0071320.s001.tif]

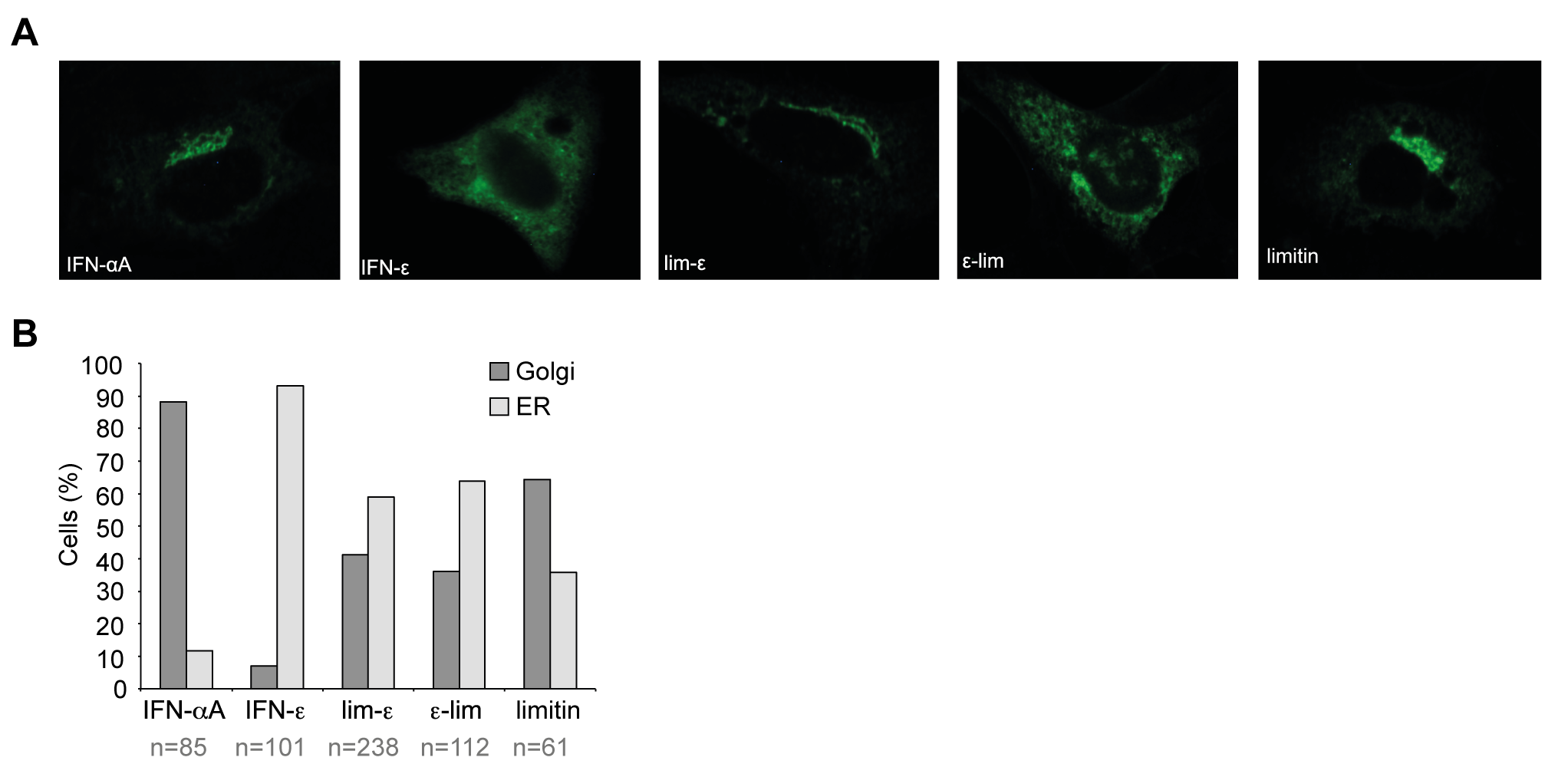

Supplement: Figure S2 — Poor progression of IFN-ε through the secretory pathway of transfected MEFs/T. A. Immunofluorescent detection of FLAG-tagged IFNs in MEFs/T cells transfected with plasmids expressing the indicated tagged IFNs. B. Histograms showing, for the indicated constructs, the proportion of cells where IFN colocalizes mostly with the Golgi (dark grey) or with the endoplasmic reticulum (light grey). The amounts of counted cells are indicated below each plot. (TIF) [file pone.0071320.s002.tif]
