# Supplementary material for: IFN-ε Is Constitutively Expressed by Cells of the Reproductive Tract and Is Inefficiently Secreted by Fibroblasts and Cell Lines
Source: PLoS One. 2013 Aug 9;8(8):e71320. doi: 10.1371/journal.pone.0071320 (PMC3739789; doi:10.1371/journal.pone.0071320)
Supplement: Materials and Methods S1 — (DOCX) [file pone.0071320.s003.docx]

**Supplemental information**

**Supplementary Materials and Methods**

Cells

Mouse embryonic fibroblasts (MEFs) were isolated from C57BL/6 mice by standard procedures. Briefly, embryos were harvested at day 14.5 of gestation. The head and organs were removed and the rest of the embryo was placed in a Petri dish containing Trypsin-EDTA (Lonza, 170 000 U/L Trypsin, 200 mg/L EDTA) in which the tissue was minced. After 13 minutes of incubation at 37°C, the tissue was homogenized by pipetting then centrifuged to eliminate undissociated tissue fragments. Cells were then grown in Dulbecco Modified Eagle medium (DMEM, Lonza ref 12-604F) containing ultraglutamine and 4.5 gr/L of glucose, and supplemented with 10% of fetal calf serum (Sigma) and 50 units/ml of penicillin/streptomycin (Lonza). MEFs were then immortalized by transduction of pPH51, a retroviral vector derived from pQCXIN (Stratagene) and expressing the simian virus 40 large T antigen. Immortalized MEFs were called MEFs/T. Transfection of these cells was performed using the LT1 reagent (Mirus), according to the manufacturer's instructions.

**Supplementary figures**

**Figure S1. *Oasl2* expression level is elevated in uterus and ovaries**

RT-qPCR data showing the expression of *Oasl2* in organs collected from uninfected female C57BL/6 mice (same as in Fig 2A). Each column refers to an individual sample and indicates the number of *Oasl2* cDNA copies per 10^2^ β-actin cDNA copies.

**Figure S2. Poor progression of IFN-ε in the secretory pathway of transfected MEFs/T**

A. Immunofluorescent detection of FLAG-tagged IFNs in MEFs/T cells transfected with plasmids expressing the indicated tagged IFNs.

B. Histograms showing, for the indicated constructs, the proportion of cells where IFN colocalizes mostly with the Golgi (dark grey) or with the endoplasmic reticulum (light grey). The amounts of counted cells are indicated below each plot.
